# Hematopoietic Stem Cell Niches and Signals Controlling Immune Cell Development and Maintenance of Immunological Memory 

**DOI:** 10.3389/fimmu.2020.600127

**Published:** 2020-11-26

**Authors:** Runfeng Miao, Vivian Y. Lim, Neeharika Kothapalli, Yifan Ma, Julia Fossati, Sandra Zehentmeier, Ruifeng Sun, João P. Pereira

**Affiliations:** Department of Immunobiology and Yale Stem Cell Center, Yale University School of Medicine, New Haven, CT, United States

**Keywords:** hematopoietic stem cell niches, lymphopoiesis, myelopoiesis, CXCR4, WHIM syndrome, leukemia

## Abstract

Studies over the last couple of decades have shown that hematopoietic stem cells (HSCs) are critically dependent on cytokines such as Stem Cell Factor and other signals provided by bone marrow niches comprising of mesenchymal stem and progenitor cells (MSPCs) and endothelial cells (ECs). Because of their critical roles in HSC maintenance the niches formed by MSPCs and ECs are commonly referred to as HSC niches. For the most part, the signals required for HSC maintenance act in a short-range manner, which imposes the necessity for directional and positional cues in order for HSCs to localize and be retained properly in stem cell niches. The chemokine CXCL12 and its Gαi protein coupled receptor CXCR4, besides promoting HSC quiescence directly, also play instrumental roles in enabling HSCs to access bone marrow stem cell niches. Recent studies have revealed, however, that HSC niches also provide a constellation of hematopoietic cytokines that are critical for the production of most, if not all, blood cell types. Some hematopoietic cytokines, namely IL-7 and IL-15 produced by HSC niches, are not only required for lymphopoiesis but are also essential for memory T cell maintenance. Consequently, hematopoietic progenitors and differentiated immune cells, such as memory T cell subsets, also depend on the CXCL12/CXCR4 axis for migration into bone marrow and interactions with MSPCs and ECs. Similarly, subsets of antibody-secreting plasma cells also reside in close association with CXCL12-producing MSPCs in the bone marrow and require the CXCR4/CXCL12 axis for survival and long-term maintenance. Collectively, these studies demonstrate a broad range of key physiological roles, spanning blood cell production and maintenance of immunological memory, that are orchestrated by stem cell niches through a common and simple mechanism: CXCL12/CXCR4-mediated cell recruitment followed by receipt of a maintenance and/or instructive signal. A fundamental flaw of this type of cellular organization is revealed by myeloid and lymphoid leukemias, which target stem cell niches and induce profound transcriptomic changes that result in reduced hematopoietic activity and altered mesenchymal cell differentiation.

## Introduction

The hematopoietic system is composed of a multitude of cell types with different properties and functionalities. Hematopoietic cells develop from a rare population of hematopoietic-lineage restricted stem and progenitor cells that, in adult mammals, reside and differentiate in the bone marrow. Many studies over the last several decades have revealed a collection of extrinsic factors, such as cytokines and chemokines, that are required for hematopoietic stem and progenitor cell maintenance and activation. For the most part, these factors are locally produced in the bone marrow by a collection of cells that form the HSC niche. Several excellent reviews on the topic of stem cell niches have already been published (1, 2) and is not the focus of this review. Instead, we will focus on the bone marrow niches and signals involved in hematopoietic progenitor differentiation, as well as the bone marrow niches required for long term maintenance of adaptive immune cells. We will discuss a significant body of work showing that the niches controlling HSC maintenance overlap with the niches promoting lymphopoiesis and differentiation of at least some myeloid cell subsets. Furthermore, we will also discuss how these same niches play important roles in the maintenance of memory T cell subsets and long-lived plasma cells.

## Instructive Signals and Niches Controlling Hematopoietic Cell Differentiation

Hematopoietic stem and progenitor cells differentiate into lymphoid, myeloid, erythroid and megakaryocyte lineages in response to extracellular signals, predominantly cytokines, produced in the local microenvironment. Hematopoietic cytokines generally act to promote and/or maintain commitment to a specific cell lineage (e.g. lymphoid, myeloid, etc.), to induce progenitor cell proliferation, and/or to signal progenitor cell survival. Although some stochasticity is likely involved in HSC differentiation decisions, hematopoietic cell production is overtly reduced without access to lineage-specific hematopoietic cytokines. Therefore, the cellular sources of hematopoietic cytokines define the local microenvironments, or niches, where specific hematopoietic cell lineages are normally produced. We will focus on the cellular sources of key hematopoietic lineage-specific cytokines in bone marrow and on the guidance cues that allow hematopoietic progenitor cells to access these niches.

### Lymphoid Cells and Their Niches

The lymphoid compartment is composed of B and T cell subsets (each greater than 10^8^ cells in mice) and of innate lymphoid cells (ILCs). All lymphoid subsets differentiate from lineage-restricted Common Lymphoid Progenitors (CLPs). CLPs express the interleukin (IL)-7 receptor complex formed by IL-7Rα and the common *γ* chain (3), and the large majority of lymphocyte subsets depend on IL-7/IL-7R signaling for their development and survival. Consequently, in IL-7 or IL-7Rα deficient mice, B and T lymphocytes are reduced by 10-100 fold (4–6), and ILC subsets are also significantly reduced (7). Besides IL-7, FMS-like tyrosine kinase 3 ligand (FLT3L) has also been shown to contribute significantly to lymphopoiesis in that combined deficiency in IL-7 and FLT3L results in essentially undetectable B and T lymphocyte production (8–10). In terms of IL-7/IL-7R dependency, the single exception is Natural Killer (NK) cells, which are normally produced in IL-7-deficient mice but are overtly reduced in IL-15 or IL-15 receptor-deficient mice (11–13). Although several other extrinsic factors have been found to play measurable roles in lymphopoiesis *in vitro* and/or *in vivo* (14), these cannot compensate for the absence of IL-7 or IL-15 and therefore will not be discussed here.

Both IL-7 and IL-15 act as short-range signals, necessitating proximity between lymphoid progenitor “client” cells and the cells producing them. IL-15 requires binding to its IL-15Rα chain for trans-presentation to client cells expressing the IL-15 signaling receptor heterodimer formed by IL-2Rβ and the common *γ* chain. IL-7 acts as a soluble cytokine, but its expression is very low, and some evidence suggests that it can be tethered onto the cell surface through binding to glycosaminoglycans (15). For these reasons, a niche promoting IL-7-dependent lymphopoiesis exists in the vicinity of cells producing IL-7, and for NK cells the niche must be formed by the cells producing and/or presenting IL-15.

A study using *Il15* reporter mice identified the cellular sources of IL-15 in the bone marrow as being mostly composed of CXCL12, VCAM1, and platelet-derived growth factor receptor beta (PDGFRβ)-expressing cells (16), which stands in agreement with prior studies that showed IL-15 expression in CXCL12+ stromal cells (17). Using a dual *Il7* and *Il15* reporter mouse, considerable overlap between the IL-7+ and IL-15+ mesenchymal stromal cell populations was discovered in bone marrow (16). Although insightful, these studies did not demonstrate that lymphoid progenitors were indeed dependent on IL-7 or IL-15 produced by mesenchymal stromal cells *in vivo*, as other cells, such as dendritic cells, can also produce IL-15 under inflammatory conditions (18).

Mesenchymal stem/progenitor cells (MSPCs) identified by surface expression of the Leptin receptor (LEPR) and PDGFRα/β constitute about 90% of all *Il7*-expressing cells in the bone marrow, the remaining 10% being predominantly sinusoidal endothelial cells (19). Side-by-side comparison between *Il7*-GFP knock-in reporter mice, and *Il7*-cre recombinase mice crossed with Rosa26lox-stop-lox-YFP mice, a strategy that allows for cell lineage tracing of *Il7*-producing cells and *Il7*-past producer cells, also revealed that *Il7*-expressing MSPCs differentiate into osteolineage cells, such as osteoblasts and osteocytes, that do not express *Il7*. Conditional *Il7* deletion in MSPCs resulted in significant reduction of IL-7-dependent B-lineage-committed CLP numbers, B cell progenitors, and overall reduced B cell production, whereas conditional *Il7* deletion in endothelial cells caused a small but significant reduction in proB and preB cell numbers that did not impact the overall size of the B cell compartment. Importantly, MSPC differentiation into osteolineage cells coincides with halted *Il7* expression, and thus, *Il7* deletion from mature osteoblasts and osteocytes has no quantitative impact on B cell production (19). It should be noted that these findings diverge from prior studies proposing that osteolineage cells form a major niche for lymphopoiesis. However, these prior studies relied on mouse models in which osteolineage cells were selectively modified or ablated by conditional gene targeting approaches that presumed to be selective in osteoblasts but that are now known to act in MSPCs with variable efficiency. In depth analyses of these studies have recently been reviewed (2, 14).

An interesting feature of IL-7-producing MSPCs is that these cells express the highest amounts of CXCL12 in bone marrow (19). CXCR4, the CXCL12 receptor, attracts not only HSCs but also lineage-restricted hematopoietic progenitor cells, such as multipotent progenitor cells (MPPs), CLPs, and early B-lineage progenitor cells to the vicinity of MSPCs (19–21). HSCs require CXCR4/CXCL12 for long-term maintenance not only because CXCR4 signaling promotes HSC quiescence directly (20, 22–24) but also because it may enable cells to encounter Stem Cell Factor (SCF, encoded by *Kitl*). Consistent with this possibility, CXCL12+ cells and SCF+ stromal cells overlap by more than 99%, and conditional *Kitl* or *Cxcl12* deletion from MSPCs result in similar phenotypes: significant reductions in HSC numbers and hematopoietic reconstitution capacity (25–27). While direct evidence of reduced ckit signaling in CXCR4-deficient HSCs is still lacking. there is evidence supporting this model in downstream hematopoietic progenitors. Specifically, *Cxcr4* conditional deletion in MPPs, or in CLPs, resulted in impaired lymphopoiesis due to a significant reduction in IL-7 receptor signaling, as measured by STAT5 phosphorylation (19). CXCR4 deletion at the B-lineage-committed proB cell stage also reduces developing B cell numbers in the bone marrow due to their premature mobilization into the periphery (28–30), possibly in combination with reduced IL-7R signaling at the IL-7-dependent proB and preB cell stages. Likewise, CXCR4/CXCL12 plays essential roles in NK cell development as it presumably guides NK progenitors toward IL-15 niches formed by MSPCs (17).

But, CXCR4 signaling plays other roles than acting as a chemoattractant receptor. For example, CXCR4 signaling promotes conformational changes in the integrin heterodimer α4β1 (VLA-4) that result in its transactivation and adhesion to fibronectin and VCAM-1 (31, 32). Hematopoietic stem and progenitor cells, and lymphoid progenitors, are exquisitely dependent on α4β1 signaling for movement and retention within the bone marrow. Defects in integrin-mediated adhesion reduce hematopoietic stem and progenitor differentiation in bone marrow, in part due to their premature release into the bloodstream (29, 30, 33, 34). Furthermore, *in vitro* studies suggested that CXCR4 may also act as a signaling receptor capable of influencing cell decisions. CXCR4 signaling drives HSC proliferation directly *via* transcriptional control of cyclin D1 and MAD1 (23), and in preB cells, CXCR4 signaling activates ERK to facilitate *Igk* germline transcription (35).

Recent single cell transcriptomic analyses of non-hematopoietic bone marrow cells have provided unprecedented resolution of MSPC clusters and differentiation trajectories during homeostasis and leukemia (36–38). Collectively, these studies not only reinforce previous findings with *Cxcl12*, *Kitl*, *Il7*, and *Il15* reporter mice described above, but also expand our knowledge of the repertoire of hematopoietic cytokines that are expressed by MSPCs and ECs in the bone marrow. For example, we now know that *Flt3l*, *Csf1*, and *Il34*, important myeloid cytokines, are primarily expressed by MSPC clusters that also express *Il7*, *Il15* and *Cxcl12* (37). Combined, these data support a model in which MSPCs not only control HSC maintenance and lymphopoiesis but may also control the development of essential myeloid cell subsets.

### Myeloid Cells and Their Niches

Most myeloid cells develop from lineage-restricted common myeloid progenitors (CMPs), with the notable exception of megakaryocytes, which may have alternative developmental pathways (39), and mast cells, for which CMP and granulocyte-monocyte progenitors (GMP)-dependent and -independent pathways have been reported (40–44). In the classical model of hierarchical hematopoietic cell development, CMPs undergo differentiation into two major intermediate progenitors, GMPs and megakaryocyte-erythroid progenitors (MEPs), both of which retain cKit expression (the SCF receptor) at relatively high levels. Defects in *Kit* (encodes cKit) or in *Kitl* ultimately lead to reduced myelopoiesis *in vitro* and *in vivo* (45), but given the critical role this pathway plays in HSC maintenance, it is difficult to separate these effects from specific roles in myeloid progenitor maintenance and/or differentiation.

Phagocytes, such as neutrophils, basophils, eosinophils, and monocytes, differentiate from highly proliferative GMPs in response to cytokines such as macrophage colony-stimulating factor (M-CSF, encoded by *Csf1*), granulocyte-macrophage colony-stimulating factor (GM-CSF, encoded by *Csf2*), and granulocyte colony-stimulating factor (G-CSF, encoded by *Csf3*). *Csf1* and *Il34*, which can signal through the M-CSF receptor (46), are critical for the development of monocyte-lineage cells *in vivo*, including bone resorbing osteoclasts (47–49), and as mentioned earlier, are both primarily expressed by bone marrow MSPCs and some ECs. Like SCF, M-CSF can be produced in soluble and membrane-bound forms (50). Therefore, M-CSF-dependent cells likely require physical proximity to the cellular niches producing it. Consistent with this possibility, transgenic expression of the membrane-bound form of M-CSF in M-CSF-deficient op/op mice partially restores the development of several monocytic cell lineages including some tissue-resident macrophage populations and osteoclast progenitors (51, 52). Given the fact that CXCL12 is the most abundant chemoattractant produced by M-CSF-expressing cells, it is interesting to note that CXCR4 expression distinguishes early and proliferative M-CSF-dependent monocyte progenitor stages from the more mature inflammatory monocyte stage (53). Importantly, conditional CXCR4 deletion in MPPs resulted in significant reduction in inflammatory monocyte development (Miao et al. in preparation), which suggests that CXCR4/CXCL12 plays a role in localizing monocytic cells in the proximity of monopoietic cytokines. In contrast to monocytic differentiation, osteoclast differentiation likely requires movement away from M-CSF niches (MSPC/EC) toward sources of RANKL produced by osteolineage cells (54–56).

In adult mice, most dendritic cell (DC) subsets differentiate from a common monocyte/dendritic cell progenitor in response to two cytokines: M-CSF and FLT3L (49, 57). Although the bone marrow niches supporting DC development and DC lineage choices have not been functionally defined, the fact that both cytokines are predominantly expressed by MSPCs and some ECs in bone marrow suggests an overlap with HSC niches. Consistent with this model, plasmacytoid DCs depend on CXCR4/CXCL12 for development in bone marrow (58). Whether conventional or monocyte-derived DC subsets are also dependent on CXCR4 remains unclear.

Due to their very short life span (6-8 h in the resting state), neutrophils are the most abundantly produced granulocyte population with an estimated rate of 5-10 x 10^10^ cells/day (59). G-CSF is the major cytokine promoting neutrophil development during both homeostasis and emergency states (59, 60). G-CSF acts at multiple stages during neutrophil development, starting at the GMP stage. A recent study examining GMP localization in bone marrow tissue noted that while GMPs are seemingly scattered throughout the bone marrow parenchyma under homeostasis, upon myelosuppressive treatment and leukemia, GMPs undergo proliferative bursts in large clusters around perivascular niches surrounded by lineage+ cells (61). GMP expansion during regeneration and leukemia was fueled by G-CSF, presumably secreted by sinusoidal endothelial cells, and by IL-1. The mechanisms of GMP clustering remain unstudied, but it is likely that CXCR4/CXCL12 plays some role in this process. Under homeostatic conditions, conditional CXCR4 deletion in MPPs (bypassing HSCs) reduced CMP, GMP and granulocyte production (19), which suggests that myeloid progenitor localization is important for myelopoiesis *in vivo*. Paradoxically, during systemic inflammation and infection, CXCL12 production is sharply decreased at the mRNA and protein levels along with reductions in lymphopoietic cytokine production (62, 63). These changes in CXCL12 levels primarily reduce the retention and production of lymphoid lineage cells in the bone marrow, presumably to allow for the expansion of short-lived myeloid cells required for protective immunity and return to homeostasis. However, reduced CXCL12 production would be expected to also reduce neutrophil (and monocyte) production (19). Nevertheless, G-CSF production is dramatically increased in the early stages of the systemic inflammatory response (64–66), which likely compensates for the negative effects of lower CXCL12 levels.

As for other granulocytes, the niches (and rules) controlling their development are less well-defined. Elegant studies using mice with thymic rudiments devoid of hematopoietic function (due to a mutation in the transcription factor *Foxn1*) showed that adding back SCF is sufficient to support mast cell differentiation *in vivo* (67), in agreement with prior *in vitro* studies (68). Whether this is also the case in the bone marrow microenvironment remains unknown. In contrast, SCF alone is insufficient for basophil and eosinophil development, as these phagocytes require specific instructive cues for their development. Basophil development is still poorly understood, but well-controlled *in vitro* studies demonstrated that combinations of SCF and IL-3, the latter secreted by mast cells, promote their development (68). Therefore, it is reasonable to consider that cellular circuits formed by granulocyte progenitors, mast cells and SCF/CXCL12+ niches in bone marrow generate an appropriate environment for basophil development. Eosinophil development is characterized by an intermediate eosinophil progenitor stage marked by low cKit and high IL-5Rα expression, and is largely, but not entirely, dependent on IL-5 (69). In contrast to most hematopoietic cells, however, the majority of eosinophils do not differentiate in the bone marrow. Instead, they differentiate from eosinophil progenitors within peripheral tissues in response to IL-5 produced locally and primarily by ILC2 cells (70–72).

### Niches for Megakaryocytes and Erythrocytes

While most hematopoietic cell lineages develop through well-defined stages, megakaryocyte development can take multiple pathways, including direct differentiation from HSC-like populations (39). Like all hematopoietic cell subsets, megakaryocyte development requires a combination of extrinsic cytokine signals for progenitor proliferation and differentiation into the final polyploid state. Early studies showed that thrombopoietin (encoded by *Thpo*) is the major cytokine that promotes megakaryopoiesis (73), and a recent elegant study showed that hepatocyte-derived, but not MSPC-derived thrombopoietin is essential for HSC maintenance and megakaryocyte development *in vivo* (74). However, the effects of thrombopoietin synergize with SCF in inducing megakaryocyte progenitor expansion and differentiation (75, 76). Whether such synergy can only occur in bone marrow niches or is also efficient in extramedullary sites is not entirely clear. Studies using CXCR4-deficient mice lend support to a model where megakaryocyte progenitor localization and differentiation takes place preferentially in bone marrow niches (77). Consistent with this model, CXCR4 expression is increased during megakaryocyte development (78), and these cells localize in perivascular niches in proximity to HSCs (79–81). In a similar manner, erythropoiesis relies on erythropoietin in synergy with SCF, which together coordinate the development of early uncommitted hematopoietic progenitors into erythroid lineage-committed developmental stages (82). Given the dependency on cKit/SCF it is not surprising that CXCR4/CXCL12 deficiency in the hematopoietic lineage also leads to erythropoietic defects (77, 83, 84).

### Bone Marrow Niches Supporting Adaptive Immunity

Besides their critical function in HSC maintenance and hematopoietic progenitor differentiation, bone marrow niches formed by CXCL12+ cells are also instrumental for ensuring the longevity of adaptive immunity. Here, we will focus specifically on two major adaptive immune cell populations, plasma cells and memory T cell subsets.

### Plasma Cell Niches

Plasma cells (PCs) are essential for humoral immunity against infections and can be divided into two sub-populations: short-lived and proliferative plasmablasts, and long-lived and quiescent plasma cells (LLPCs). A large fraction of LLPCs reside in bone marrow and provide the body with long-term protection *via* constitutive antibody production (85–87). Besides the bone marrow, LLPCs also reside in secondary lymphoid organs, the gastrointestinal tract, and other mucosal-associated tissues (88). The microenvironments where LLPCs reside are thought to play important roles in LLPC survival because these cells lack the intrinsic ability to survive in the absence of extrinsic factors (89, 90). LLPC survival factors include ligands for the TNF superfamily member BCMA (B cell maturation antigen, gene symbol *Tnfrsf17*), namely B cell activation factor (BAFF, encoded by *Tnfsf13b*) and a proliferation-inducing ligand (APRIL, encoded by *Tnfsf13*), several cytokines of which IL-6 plays a prominent role, costimulatory B7 family members CD80 and CD86, CD44, CXCL12, and adhesion receptors (91).

PC survival factors can be divided into two groups: signals that directly control the expression of anti-apoptotic molecules (e.g. *Bcl2* family members) and factors that control PC localization in appropriate niches (e.g. chemokines, adhesion receptors). Of the signals that directly promote PC survival, BCMA is perhaps the most impactful as BCMA-deficient mice show the largest reduction in antigen-specific PC numbers in the bone marrow (92, 93). Consistent with such a prominent role, BCMA signaling induced by BAFF or APRIL promotes the expression of the anti-apoptotic *Bcl2* family member, *Mcl1* (93, 94). Interestingly, the dependence on BCMA for PC survival is tissue-specific, as BCMA does not control the expression of *Mcl1* or other *Bcl2*-family members in splenic PCs, and BCMA-deficiency does not reduce splenic PC numbers (93).

BAFF and APRIL bind to BCMA (95, 96) with APRIL binding with higher affinity (97, 98). The loss of both signals impairs PC survival in bone marrow (94, 96). BAFF is produced by myeloid precursors and neutrophils (99, 100), and APRIL is expressed by myeloid precursors, monocytes/macrophages, eosinophils and megakaryocytes in bone marrow (99–103). PCs have been found in close proximity with cells expressing BCMA ligands in bone marrow, and genetic models of megakaryocyte or eosinophil deficiency revealed small but significant reductions in bone marrow PC numbers (99–103). Besides BCMA ligands, myeloid cells may also provide other soluble and membrane-bound PC survival signals such as CD28 ligands and cytokines, which in the case of IL-6 can synergize with APRIL or other soluble factors secreted by stromal cells to extend PC survival (104). In turn, *in vitro* studies showed that interactions between PCs and stromal cells induced further IL-6 production (89). Similarly, secretion of IL-6 by DCs can also be induced by interactions with PCs through CD28-CD80/CD86 (105). These findings suggest that positive feedforward mechanisms driven by complex cellular interactions between PCs and niche cells may operate *in vivo* in a manner reminiscent of cell circuits between proB cells and IL-7-producing MSPCs (14, 21). However, it should be noted that very few MSPCs, osteolineage cells, and endothelial cells in bone marrow express IL-6 *in vivo* under homeostatic conditions (37), and that single deficiency in *Il6* does not cause measurable reductions of PC numbers *in vivo* (90).

Although some PCs may be able to differentiate in the bone marrow environment (106, 107), the large majority of PCs differentiate from activated B cells in secondary lymphoid organs and require a coordinated change in chemoattractant responsiveness to migrate into the bone marrow (108, 109). CXCR4 in particular is essential for PC homing into the bone marrow (110) and once inside this compartment, PCs localize adjacent to CXCL12-expressing cells (111) where they remain relatively static over short periods of time (112). The mechanisms underlying this non-motile behavior have not been elucidated but it is likely the result of high CXCR4 signaling in combination with integrin-mediated adhesion, namely α4β1- and αLβ2-mediated interactions with fibronectin and ICAM1 expressed on CXCL12-producing cells (89, 113). Signaling induced by αLβ2 and α4β1 activation may also prevent apoptosis *via* the phosphatidylinositol-3-kinase (PI3K)-protein kinase B (AKT) pathway (114, 115). Besides myeloid lineage cells, perivascular DCs also localize in CXCL12+ niches (116) and may contribute to PC survival *via* direct delivery of CD80 and CD86 signals (105). Combined, these studies demonstrate that a complex network of cells and signals promote PC survival and suggest that PC localization in bone marrow niches is a pre-requisite for receiving survival signals.

But, can PCs destined to reside in bone marrow niches survive elsewhere? The answer varies by the type of PC. Under conditions of acute immunization with model antigens, PCs specific for T-independent antigens depend on CXCR4 for homing into the bone marrow. Accordingly, defects in this process result in decreased PC numbers in bone marrow and reduced antigen-specific IgM and IgG production. PCs developing from T-dependent antigens from acute immunizations also require CXCR4 for bone marrow homing, but serum antigen-specific IgM, IgG and IgA concentrations remain normal (28). These data lend strong support to a model where LLPCs generated from acute immunizations destined for bone marrow niches can survive in peripheral sites and secrete antigen-specific antibodies for extended periods of time (117). Hence, PC localization in bone marrow niches may play other roles that are independent of antibody secretion (118, 119).

### Bone Marrow Niches for T Cells

Most lymphoid organs contain large numbers of naïve T cells due to the activity of transcriptional programs controlled by Krüppel-like Factor 2 (KLF2), which coordinates the expression of a key set of genes responsible for T cell trafficking (120, 121). Naïve T cell re-circulation is essential for naïve T cell survival due to the fact that these cells depend on external signals, such as IL-7 and Sphingosine 1-phosphate (S1P), for homeostatic survival (122, 123). While both of these signals could theoretically be provided in bone marrow niches, naïve T cells seldom migrate into this compartment due to very low CXCR4 expression, a process that is actively regulated by Sin1-dependent mammalian target of rapamycin complex-2 (mTORC2) signaling and FOXO1 inactivation (124). The signals upstream of mTORC2-FOXO1 remain undefined. The bone marrow is, however, a reservoir for some T cell subsets. Long-lived CD4+ and CD8+ central memory (CM) T cells undergo cytokine-driven homeostatic self-renewal to ensure long-term maintenance of the memory T cell pool. CM CD8+ T cells mainly depend on IL-15 for homeostatic self-renewal, whereas CM CD4+ T cells are more dependent on IL-7 than IL-15 (125, 126). Even though sources of IL-7 and IL-15 exist in multiple organs throughout the body (16, 127–129), as previously discussed, both cytokines are also produced by bone marrow MSPCs (37). Importantly, CXCR4-mediated homing to the bone marrow is essential for the survival of CM T cells (130–133).

Given the fact that CM lymphocytes reside in the bone marrow and share niches with HSCs and their descendants, mechanisms have evolved to keep antigen-experienced lymphocytes under control. Regulatory T cells (Tregs), a T cell population that is essential for immune tolerance, seem to play such roles in bone marrow niches. Under homeostatic conditions, Tregs account for 20% to 40% of CD4+ T cells in bone marrow. Bone marrow Tregs differ from peripheral Tregs in their increased ability to express IL-10 and the checkpoint receptor CTLA-4 (134). This is particularly relevant in bone marrow transplantation, where Tregs have been described to promote tolerance to allogeneic HSCs (135). In the scurfy mouse model, Treg deficiency results in profoundly abnormal hematopoiesis (136), although it is difficult to separate local effects in the bone marrow microenvironment from systemic inflammation and the ensuing emergency myelopoiesis (60, 62, 63). Besides their role in maintaining peripheral tolerance, some evidence suggests that Tregs can also directly control the homeostasis of several hematopoietic cell lineages. For example, bone marrow Tregs promote HSC quiescence *via* adenosine secretion (137), although it is unclear if this is through direct crosstalk with HSCs, or through indirect effects on CM and other T cell subsets. Tregs also promote LLPC survival and osteoclast differentiation through CTLA-4 (134). The fact that Tregs maintain a state of immune privilege in bone marrow for the homeostasis of HSCs and other hematopoietic cells provides another elegant example of the multifunctionality of HSC niches in bone marrow.

## Lessons From WHIM Syndrome

A key feature of most cellular receptors is their ability to undergo ligand-induced receptor internalization and signal termination. This is particularly relevant for physiological control of chemoattractant receptor function as it allows cells to desensitize from one chemoattractant and respond to other cues emanating from adjacent locations (138, 139). More than 95% of hematopoietic cells in bone marrow express CXCR4 and migrate toward sources of CXCL12. Most, if not all, hematopoietic cell lineages utilize the CXCR4/CXCL12 pathway during development from HSCs and downstream multipotent progenitors. Like most chemoattractant receptors, CXCR4 is internalized upon binding to CXCL12, and CXCR4 desensitization is an important mechanism of controlled hematopoietic cell exit from the bone marrow (33). Defects in CXCR4 desensitization alone can cause numerous physiological defects in hematopoietic cell development and recirculation that result in immune deficiency, as evidenced by patients afflicted with WHIM syndrome.

WHIM syndrome is an extremely rare combined immunodeficiency disorder caused predominantly by heterozygous nonsense and missense mutations in the cytoplasmic tail of CXCR4, the most common being the replacement of Arginine 334 by a stop codon, which deletes the last 19 amino acids of the C-terminus domain (140, 141). The cytoplasmic domain controls CXCR4 desensitization by recruiting GPCR kinases (GRKs) followed by phosphorylation of serine/threonine residues and β-arrestin recruitment (142, 143). Therefore, mutations in the cytoplasmic tail of CXCR4 are typically gain-of-function, resulting in increased CXCR4 signaling in response to its ligand CXCL12 (144). The frequency of WHIM syndrome is estimated to be 0.23 per million births (145). The WHIM acronym is defined by disease symptoms: **W**arts, **H**ypogammaglobulinemia; **I**nfections, which are common in the respiratory and mucosal tracts, patients being particularly susceptible to Human Papilloma Virus infections; and **M**yelokathexis, the retention of neutrophils in bone marrow that is responsible for very low neutrophil counts in peripheral blood. Most patients also present with peripheral blood panleukopenia, particularly B lymphopenia and a paucity of plasmacytoid dendritic cells (Majumdar and Murphy, 2018).

Given the dominant effects of CXCR4/CXCL12 at multiple stages of hematopoietic cell development, gain-of-function mutations in CXCR4 are also expected to impact blood cell production. A mouse model of WHIM syndrome generated by Balabanian and colleagues reproduced several hematological defects seen in WHIM patients, including peripheral blood leukopenia, that could be reversed by CXCR4 antagonism (146). Likewise, WHIM patients treated with a low dose of a CXCR4 antagonist also restore leukocyte numbers in peripheral blood and have reduced susceptibility to infectious diseases (147). In the WHIM mouse model, peripheral blood leukopenia was mostly caused by reduced B cells and neutrophils. Besides mature B cells, CD4 and CD8 T cells were also significantly reduced in the spleen, suggesting defective B and T cell development in primary lymphoid organs. In contrast, neutrophil development was seemingly intact, with mature neutrophils being somewhat increased in the bone marrow perhaps as a consequence of reduced egress from this compartment (146).

A combination of serendipity and careful molecular and cellular studies of a single WHIM patient revealed surprising insights into the delicate balance between HSC quiescence, activation, and multilineage differentiation. Identified at the NIH as patient WHIM-09, this patient spontaneously cured leukopenia and susceptibility to infectious diseases, due to a chromothripsis event in chromosome 2 of presumably a single hematopoietic progenitor cell, which eliminated the *Cxcr4^R334X^* allele along with 163 other genes and switched a hyper-responsive for an haploinsufficient CXCR4 state (148). Studies in mice showed competitive advantage in hematopoietic reconstitution of HSCs haploinsufficient for CXCR4, thus providing a plausible explanation for why chromotryptic “cure” of *Cxcr4^R334X^* allele in a single hematopoietic progenitor resulted in the replacement of leukocytes expressing the R334X mutant CXCR4 by the single wild-type allele (148). Paradoxically, in the WHIM-09 patient, lymphoid cells only differentiated from R334X-expressing hematopoietic progenitors, while myeloid cells differentiated from hematopoietic progenitors expressing wild-type CXCR4, albeit at reduced amounts due to CXCR4 haploinsufficiency. The fact that short-lived myeloid cells such as neutrophils are only produced by CXCR4 wild-type expressing hematopoietic progenitors decades after chromothripsis occurred strongly suggests that deletion of the *Cxcr4^R334X^* allele occurred in a single HSC, which expanded and self-renewed over time. However, this model is not easily compatible with the fact that lymphoid lineage cells develop from hematopoietic progenitors carrying *Cxcr4^R334X^* alleles. One possibility is that one or several of the 163 genes deleted are directly or indirectly critical for lymphopoiesis. Alternatively, expression of hyper-responsive R334X CXCR4 in a few hematopoietic progenitors diluted in a sea of hematopoietic stem and progenitor cells expressing 50% of wild-type CXCR4 confers a very strong competitive advantage for contacts with niche cells providing lymphopoietic factors (19, 21). It should be noted that lymphoid progenitors are particularly sensitive to efficient CXCR4 desensitization for their proper development from hematopoietic progenitors (149), and thus studies are needed to fully understand how changes in CXCR4 signaling intensity translates into alterations in hematopoietic cell lineage decisions.

## Leukemia and Its Impact on Bone Marrow Niches, Hematopoiesis, and Immune Cells

The tight regulation over cell proliferation, differentiation, and quiescence carried out by the HSC niche is severely disrupted in the context of malignancy (150). Studies over the last few years have revealed that several types of blood cancers interact with MSPCs and ECs and alter their ability to produce homeostatic cytokines and chemokines. Mouse models of chronic myelogenous leukemia (CML) showed significant reductions in the expression of HSC niche factors, including *Cxcl12*, *Lepr*, *Kitl* and *Angpt1*, and concomitant expansion of osteolineage cells (151, 152). The expansion of osteolineage cells contributed to bone marrow fibrosis, a phenomenon also observed in CML patients (153). These observations have been reinforced by single-cell RNA-sequencing of stromal and endothelial cells of mice transplanted with acute myeloid leukemia (AML), which revealed a block in adipogenic and osteogenic differentiation programs in MSPCs and osteolineage cells, as well as a reduction of *Cxcl12* and *Kitl* in MSPCs and arteriolar ECs (36). In addition, *Angpt1* and *Il7* expression in MSPCs was also decreased. Furthermore, in myeloproliferative neoplasms (MPNs), hyperactivated hedgehog (HH) signaling, predominantly driven by the overproduction of HH ligands by malignant cells, results in decreased numbers of bone marrow MSPCs and osteoblasts and the downregulation of niche-derived HSC-maintenance factors (154). Specifically, *Kitl* and *Cxcl12* are downregulated especially in endothelial cells and CXCL12-abundant reticular (CAR) cells, and *Jagged1* is downregulated in endothelial cells (154).

Besides myeloid malignancies, lymphoid malignancies have also been shown to alter the bone marrow microenvironment. An early study using adoptive transfer of the Nalm6-GFP preB Acute Lymphoblastic Leukemia (ALL) cell line found reduced *Cxcl12* expression in poorly-defined bone marrow niches, resulting in displacement of normal hematopoietic stem and progenitor cells (155). More recently, our group demonstrated that pre-malignant preB cells with unrepaired double-stranded DNA breaks induce the downregulation of *Il7* transcription in bone marrow MSPCs, while BCR-ABL preB ALL cells downregulate both *Il7* and *Cxcl12* in MSPCs *via* undefined mechanisms (21).

In addition to the reduced expression of key niche factors, the overproduction of pro-inflammatory cytokines by niche cells has also been observed in the early stages of multiple hematological malignancies. IL-1β is one of the first pro-inflammatory cytokines abnormally increased in the development of MPN and CML in mice (156), and clinically, such elevated levels of IL-1β in CML patients have been associated with poor prognosis (157). Other cytokines and growth factors such as IL-6, thrombopoietin, and CCL3 have also been suggested to render the bone marrow microenvironment pro-inflammatory in AML, ALL, MPN, and CML (152, 156, 158, 159).

This pro-inflammatory milieu disrupts normal HSC niche function primarily in two ways. First, it damages sympathetic nerve fibers that innervate arterioles, which are essential for maintaining MSPC quiescence (156, 160). In AML, sympathetic neuropathy has been correlated with skewed Nestin (gene symbol *Nes*)-producing niche cell fate determination, wherein MSPCs primed for the osteoblastic lineage significantly expand at the expense of HSC-maintaining arteriole-associated Neuron-glial antigen 2 (NG2)-expressing niche cells (161). Second, the inflammatory signals, possibly in combination with HH signaling, reduce CXCL12 expression in niche cells (151, 156, 161).

Together, these niche changes impair normal hematopoiesis while favoring the growth of leukemic malignancies (21, 161–163). In AML patients, total hematopoietic progenitors (CMPs, GMPs and MEPs) are reduced (164), and likewise, bone marrow samples from pediatric cases of ALL have lower levels of myeloid progenitor cells and erythroid progenitor cells than control samples (158). One possible explanation for the selective growth of leukemic cells is that other pathways could complement the CXCR4/CXCL12 axis in malignant cells to enable their migration. For instance, some studies have shown that Bruton’s Tyrosine Kinase (BTK) signaling is important for mediating leukemic cell migration toward CXCL12 in chronic lymphocytic leukemia and multiple myeloma (165, 166). As such, malignant cells may have an advantage in the competition for niche occupancy when CXCL12 concentration becomes limiting. Furthermore, other studies have shown that as leukemia progresses, leukemic cells become less dependent on certain niche factors than normal hematopoietic cells. For example, while pre-leukemic stem cells (LSCs) in AML are highly dependent on niche-derived Wingless-type (Wnt) signals in a manner similar to long-term HSCs, established LSCs and AML are unresponsive to Wnt inhibitors due to cell-intrinsic activation of Wnt signaling (167). Hence, AML cells downregulate niche factors to which normal hematopoietic cells are more sensitive, possibly as a method to gain a competitive advantage.

On the other hand, defects in the bone marrow microenvironment can themselves initiate the development of hematopoietic malignancies (150). Activating mutations of the protein tyrosine phosphatase SHP2 in MSPCs marked by *Nes*-cre, *Prx1*-cre, *Lepr*-cre or *Osx*-cre all result in MPNs, in part due to excessive production of CCL3 by MSPCs (168). In addition, deletion of Dicer1 in *Osx*-cre-expressing MSPCs led to myelodysplasia and even AML in a small percentage of mice (169). In this case, Dicer1 deficiency resulted in reduced expression of Sbds, the gene that is mutated in Schwachman-Bodian-Diamond syndrome, a human bone marrow failure and leukemia pre-disposition condition. Importantly, deletion of Sbds using *Osx*-cre also led to myelodysplasia. In other studies, Sipa1 and retinoic acid receptor gamma (RAR*γ*) deficiency in radio-resistant cells led to the development of myeloproliferative syndromes (170, 171). Interestingly, an activating mutation of beta-catenin in mouse osteoblasts led to the development of AML, where wild type hematopoietic stem and progenitor cells acquired chromosomal aberrations and the ability to propagate disease autonomously even after transplantation into a wild type environment (172). This demonstrates that dysregulation of the bone marrow niche can even enable the transformation of mutant hematopoietic cells.

In summary, leukemic cells can cause profound transcriptional changes in critical bone marrow niche cells, though the molecular mechanisms underlying these alterations remain poorly defined. It is possible that the molecular crosstalk between malignant cells and the bone marrow niche gradually remodels normal niche behavior to foster leukemic growth and attenuate normal hematopoiesis. Also poorly understood is whether leukemias affect the long-term maintenance of adaptive immune cells that require access to critical bone marrow survival niches, though recent studies suggest that may be the case (173, 174).

## Concluding Remarks and Unanswered Questions

The bone marrow niches formed by MSPCs and endothelial cells provide an array of soluble and membrane-bound cytokines and chemoattractants that not only control HSC maintenance but also support hematopoietic progenitor commitment into multiple hematopoietic cell lineages. Besides orchestrating unperturbed hematopoiesis, these niches also contribute to long-term maintenance of immunological memory through the production of key homeostatic cytokines such as IL-7 and IL-15. These observations raise a number of physiologically relevant questions: What factors and mechanisms control the size of the HSC compartment under homeostasis? Is there direct competition between HSCs, hematopoietic multipotent progenitors, and adaptive immune cells for common factors, and if so, how is homeostasis of each cellular compartment achieved? HSCs, hematopoietic progenitors, plasma cells and memory T cells are critically dependent on CXCR4 for bone marrow homing, and therefore it is likely that these cells compete for proximity or even direct contact with niche cells. Consistent with this model, CXCL12 production is highest in MSPCs producing critical HSC maintenance factors and cytokines. However, there is evidence indicating that HSC niches are not saturated and can sustain up to 2- to 3-fold increased numbers of HSCs for extended periods of time (175). It is possible that heterogeneous production of additional chemoattractants may control the differential localization of HSCs, hematopoietic progenitors, and adaptive immune cells in the proximity of distinct niche cells. Future studies are needed to further examine the intricate relationship between HSCs, hematopoietic progenitors, and terminally differentiated immune effector cells. The recent advances in HSC visualization *in vivo* should allow these questions to be addressed (34, 176).

An increasing body of work strongly indicates that the production of hematopoietic cytokines and chemoattractants by HSC niche cells is regulated by soluble inflammatory cytokines (e.g. TNFα, IL-1β), but may also be regulated by other cues provided by leukemic cells. The fact that inflammation induces a switch in blood cell production from homeostatic and balanced lymphoid and myeloid production to an emergency state of increased myelopoiesis suggests that these distinct cell lineages compete for limiting factors in the local microenvironment. Leukemic cells and non-leukemic hematopoietic cells are also likely to compete for certain types of factors (e.g. anabolic nutrients) such that leukemic cells may exploit hard-wired mechanisms of cytokine/chemokine production to gain competitive advantage by reducing the fitness of non-leukemic hematopoietic progenitors. Furthermore, it is still unclear if leukemia or inflammation-induced changes in cytokine/chemokine production by individual niche cells are reversible, or if altered cytokine/chemokine production is coordinated with niche cell differentiation and/or survival. Future studies addressing these questions are likely to uncover novel mechanisms and pathways that may be applied therapeutically to reduce leukemic cell competitive advantages, to improve HSC transplantation, and to enhance the lifespan of immunological memory against pathogens.

## Author Contributions

RM, VL, and NK made extensive review of the literature listed and drafted different sections of the review. YM and JF drafted a section of the review focused on the WHIM syndrome with help from SZ. RS drafted a section of the review focused on blood cell malignancies with help from NK. JP wrote the manuscript with input from all authors. All authors contributed to the article and approved the submitted version.

## Funding

This study was funded by the NIH (grants R01 AI11304006A1 and R21AI146648).

## Conflict of Interest

The authors declare that the research was conducted in the absence of any commercial or financial relationships that could be construed as a potential conflict of interest.
